# Binpairs: Utilization of Illumina Paired-End Information for Improving Efficiency of Taxonomic Binning of Metagenomic Sequences

**DOI:** 10.1371/journal.pone.0114814

**Published:** 2014-12-31

**Authors:** Anirban Dutta, Disha Tandon, Mohammed MH, Tungadri Bose, Sharmila S. Mande

**Affiliations:** Bio-Sciences R&D Division, TCS Innovation Labs, Tata Research Development & Design Centre, Tata Consultancy Services Ltd., 54-B Hadapsar Industrial Estate, Pune 411013, Maharashtra, India; Oklahoma State University, United States of America

## Abstract

**Motivation:**

Paired-end sequencing protocols, offered by next generation sequencing (NGS) platforms like Illumia, generate a pair of reads for every DNA fragment in a sample. Although this protocol has been utilized for several metagenomics studies, most taxonomic binning approaches classify each of the reads (forming a pair), independently. The present work explores some simple but effective strategies of utilizing pairing-information of Illumina short reads for improving the accuracy of taxonomic binning of metagenomic datasets. The strategies proposed can be used in conjunction with all genres of existing binning methods.

**Results:**

Validation results suggest that employment of these “Binpairs” strategies can provide significant improvements in the binning outcome. The quality of the taxonomic assignments thus obtained are often comparable to those that can only be achieved with relatively longer reads obtained using other NGS platforms (such as Roche).

**Availability:**

An implementation of the proposed strategies of utilizing pairing information is freely available for academic users at https://metagenomics.atc.tcs.com/binning/binpairs.

## Introduction

Metagenomic studies have been instrumental in deciphering the microbial diversity of various ecosystems. Recent developments in sequencing technologies have enabled researchers to study the entire genomic content of microbial inhabitants present in any given environment (through whole genome shotgun sequencing). Building microbial community profiles from sequence data subsequently involves comparison of the sequenced DNA reads against existing databases that contain sequences from already classified/characterized organisms.

Next generation sequencing (NGS) technologies employed for metagenomics studies rely on different sequencing chemistries, thereby leading to variations in lengths of sequenced fragments (reads) as well as throughput (sequencing depth). While accurate taxonomic classification of each individual read to its source organism by various binning algorithms depends on the length of individual reads [1], understanding the complete taxonomic diversity of any metagenomic sample depends on the total number of sequenced reads (i.e. sequencing depth). Furthermore, the choice of binning approach for any metagenomic dataset is guided by the length of reads constituting that dataset. For example, performances of binning approaches based on the comparison of compositional characteristics of sequences are reliable only for longer sequences, having lengths sufficient enough to emanate a robust compositional signature. On the other hand, sequence alignment-based binning approaches are found to perform well for a wide range of read lengths. Out of the currently popular NGS technologies, while Illumina sequencers provide a high sequencing throughput and result in read lengths around 100–200 base pairs (bp), Roche-454 sequencing platform generates relatively longer reads (approximately 400–600 bp) with significantly lower throughput. ‘Paired-end’ sequencing protocol offered by some sequencing platforms (e.g., Illumina, Solid, etc.) further allow sequencing of a single DNA fragment from both its ends. Adopting such a sequencing protocol therefore allows obtaining double the volume of sequence data, as compared to standard ‘fragment-library’ based sequencing, even when the individual read lengths remain short. Short read sequencing using the paired-end protocol is becoming increasingly popular due to relatively lower running costs.

The current generation of Illumina sequencing platforms (HiSeq/MiSeq) can generate paired-reads having cumulative length of each pair around 200 bp ×2. In addition to increasing the total volume of sequenced DNA, adoption of the paired-end technology has other potential benefits in the field of metagenomics. Since the origin of both the paired-reads can be traced back to a single DNA fragment, the ‘pairing’ information is expected to improve the taxonomic classification of the reads. An *in silico* approach of utilizing the ‘pairing’ information of individual sequenced fragments for improving the accuracy and specificity of taxonomic binning has been developed and implemented in the MEGAN tool [2]–[3]. In this approach, the alignment scores (i.e. ‘Bit scores’ in the Blast output) of individual read pairs are cumulated to generate a ‘combined Bit-score’ for the concerned read pair. Utilizing such combined ‘Bit-scores’ was observed to provide taxonomy assignments with a higher level of accuracy and confidence. Improved binning efficiency was observed for simulated metagenomic datasets containing not only ‘short-clones’ (standard paired-end sequencing – wherein a single short fragment of length ∼500 bp is sequenced from either ends), but also ‘long-clones’ (mate-pair library having insert sizes ∼2 to 5 Kbp, i.e. the sequenced pairs being widely separated on the source genome). The use of this approach is however restricted to only alignment-based binning methods (more specifically to MEGAN). It may be noted here, that in spite of providing comparatively better accuracy and specificity than composition-based binning approaches [4], alignment-based approaches require exceptionally high execution time. For example, alignment-based binning of a typical (Illumina platform generated) metagenomic dataset (consisting of ∼10–100 million reads) would require several weeks, even on a high performance compute server. In contrast, a composition-based approach would be able to process similar volume of sequence data within a day or two. The binning time required by hybrid approaches like PhymmBL [5], SPHINX [6], TWARIT [7], etc., which use a combination of alignment and composition based methodologies, are also faster by several orders of magnitudes as compared to the alignment-based approaches. The present study reports alternate *in silico* strategies that can effectively utilize pairing information along with any genre of binning approaches in order to provide improved taxonomic binning accuracy.

## Methods

### ‘Binpairs’ strategies

Most taxonomic binning approaches independently classify the two DNA fragments forming a mate-pair (although both originate from a single but longer DNA fragment). In an ideal scenario, the two reads forming a mate-pair would be assigned to the same taxon. However, in cases where the ‘mates’ are assigned to different taxonomic levels, the taxonomic assignment information of one of the reads can be utilized to improve the non-specific/wrong assignment of its mate. It has been assumed that when taxonomic levels are arranged in the order of Superkingdom>Phylum> Class> Order> Family> Genus> Species, they represent a succession from a ‘higher’ (non-specific) taxonomic level to a ‘lower’ (specific) taxonomic levels.

**Figure 1:**
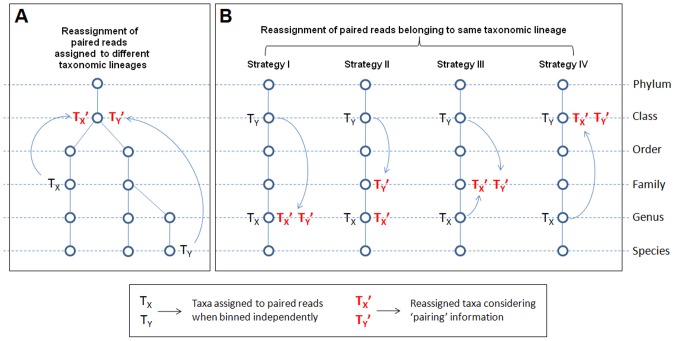
Schematic representation of different ‘Binpairs’ strategies for performing ‘taxonomic re-assignment’ of ‘paired-end’ reads. (A) Outcome when the two taxons assigned belong to two different lineages, (B) Outcome when the two taxons assigned belong to the same lineage.

In a scenario where the two reads (X and Y) forming a mate pair are assigned to different taxa (Tx and Ty) belonging different lineages, the assignments for both the reads are changed to their lowest common ancestral (LCA) taxonomic level (Fig. 1A). In scenarios where the mate-pair reads (X and Y) have been assigned at different taxonomic levels having same lineage (Fig. 1B), the following four simple ‘Binpairs’ strategies can be utilized. For illustrating the current strategies, it has been considered that the hypothetical mate-paired reads, X and Y, have been assigned to the taxonomic levels Tx and Ty respectively, where Ty> Tx. It may be noted here that although reassigning either X or Y to a lower/deeper taxonomic level means a more specific assignment, the chances of getting the assignment wrong is also higher. On the other hand, while reassigning the reads to a higher taxonomic level results in loss of specificity, it increases the probability of correct assignments.

- **‘Binpairs’ strategy I (SI):** Both X and Y are assigned to the lower taxon amongst the two, i.e. Tx. This strategy is expected to increase the specificity of the assignment, and will provide the best result in cases where the assignment of X has been accurate.
- **‘Binpairs’ strategy II (SII):** X remains assigned to Tx; Y is reassigned to a ‘lower’ and intermediate level of Tx+1 (where Tx+1< =  Ty). This strategy is also expected to increase specificity by reassigning Y to a more specific level. However in this case the reassignment of Y is slightly conservative, reducing the chance of a ‘wrong assignment’.
- **‘Binpairs’ strategy III (SIII):** Both X and Y are reassigned to the intermediate level Tx+1 (where Tx+1< =  Ty). While this strategy aims at increasing the specificity of assignment of Y (similar to Strategy II), it also moves X’s assignment to an intermediate level Tx+1, thereby reducing the probability of wrong assignment of X.
- **‘Binpairs’ strategy IV (SIV):** This is the most conservative of the 4 strategies, where X and Y are both assigned to the higher taxon amongst the two, i.e. Ty. This strategy is specifically aimed at rectifying any probable wrong assignment of X during the earlier binning step.

### Datasets for validation and testing

Each of the above described ‘Binpairs’ strategies has been evaluated to assess the improvement in specificity and sensitivity of assignments. In addition to evaluating the improvements in total number of taxonomic assignments as well as overall specificity of metagenomics binning, it is equally important to track the number of correct/incorrect assignments. The binning ‘Binpairs’ strategies mentioned above were evaluated with simulated metagenomics reads. A simulated microbiome was constructed by combining randomly picked DNA fragments (in equal proportions) from the complete genome sequences of 20 organisms (S1 Table). For comparing the efficacy of utilizing paired-end information over using only fragment reads for binning, following three sets of reads were generated from the simulated metagenome.

1. 20,000 reads with average length of approximately 400 bp, representing sequenced reads generated from a Roche 454 GS FLX platform
2. 20,000 mate pairs of lengths 2×150 bp representing ‘short-clones’ (having insert sizes of about 200 bp)
3. 20,000 mate pairs of lengths 2×150 bp representing ‘long-clones’ (having insert sizes of around 2–5 Kbp).

The ‘short-clone’ mate pairs consisted of two 150 bp fragments selected from either ends of each of the 20,000 simulated ‘Roche’ reads. The ‘long-clone’ pairs had one of the 150 bp fragments selected from a corresponding Roche read, while the other 150 bp fragment was sourced from an appropriate distance in the parent DNA molecule to allow insert sizes in the range of 2–5 Kbp. In order to mimic sequencing errors, random mutations (with an overall mutation frequency of one in every 100 bp) were introduced in all the reads belonging to these three validation sets.

Reads from a real oral metagenome [8] were used for testing. For this purpose, metagenomic sequence dataset (sample no. 4447970.3) consisting of 98000 reads, having an average read length of around 425 bp, was selected. To get an ‘equivalent’ set of reads mimicking ‘Illumina mate pairs’, 150 bp fragments were picked from both ends of the reads from the original dataset. A final set of 98000 Illumina ‘short-clones’ mate-pairs (150 bp ×2) was thus constructed. The Roche 454 reads of the original dataset, and the derived ‘Illumina-like’ mate-paired reads were subjected to binning, in order to compare and evaluate the proportion of different taxa detected from the metagenome.

### Binning programs used

Reads in the three simulated metagenomic datasets were classified using six different binning algorithms, namely, MEGAN [2]–[3], SOrt-ITEMS [9], DiScRIBinATE [10], SPHINX [6], INDUS [11] and TWARIT [7]. While MEGAN, SOrt-ITEMS and DiScRIBinATE are alignment-based approaches, INDUS rely on elucidating compositional signatures. SPHINX and TWARIT are ‘hybrid’ algorithms which utilize compositional signature based clustering steps as well as alignment-based assignment strategies. All these algorithms classify/bin sequences into taxonomic groups after comparison with an existing database of ‘known’ sequences. A database consisting of complete genomic sequences of 952 known microbial species, previously used in TWARIT, INDUS and SPHINX, was provided to all the algorithms for this purpose. In order to evaluate the specificity of assignments of reads originating from ‘unknown taxa’, it was ensured that representatives of some selected species/class/family (corresponding to some of the organisms selected for generating the simulated metagenomics datasets) were absent in this database.

## Results and Discussion

**Figure 2:**
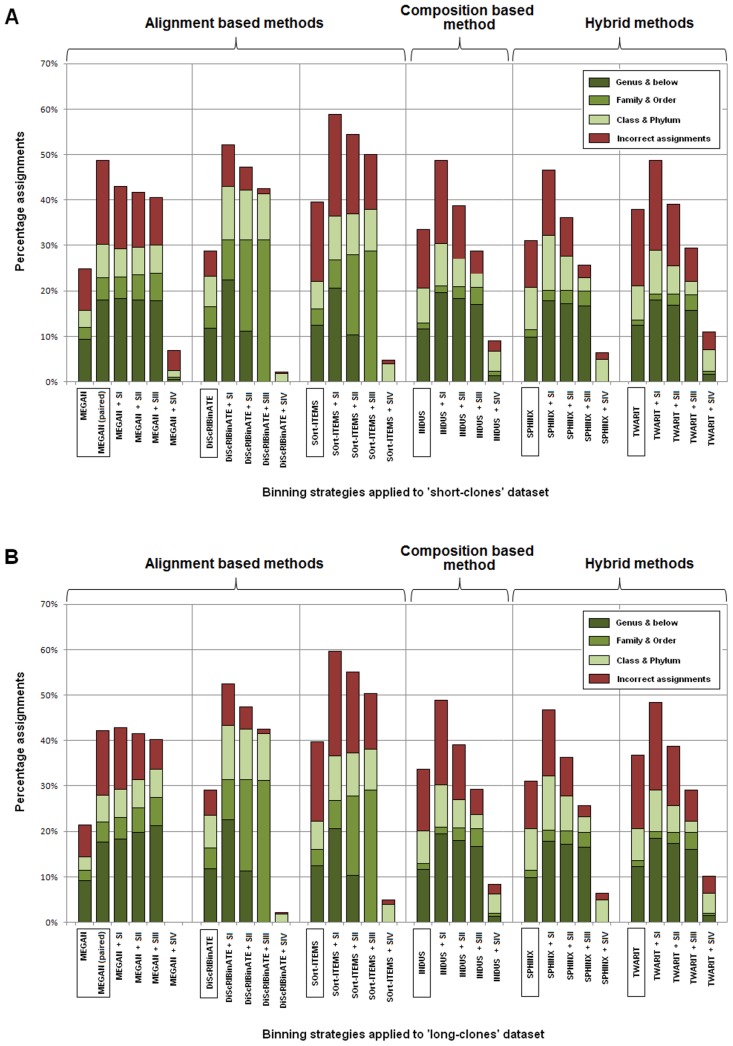
Binning outcomes for the (A) ‘short clones’ and (B) ‘long clones’ mate-paired datasets. The outcomes were the result of the application of the four ‘Binpairs’ strategies (SI, SII, SIII, SIV) in combination with different existing binning methods (MEGAN, DiScRIBinATE, SOrt-ITEMS, TWARIT and INDUS). The results obtained when reads from these datasets were binned as individual fragments (i.e. without utilizing pairing information) by the respective binning methods, are also depicted for comparison.

The binning results for both ‘short clones’ and ‘long clones’ datasets, as obtained with different binning algorithms and the proposed ‘Binpairs’ strategies, are summarized in Fig. 2. The efficiency of different methods/strategies is depicted in the figure in terms of correct taxonomic assignments (at different specific levels), wrong assignments, as well as the proportion of unassigned reads.

### Validation of ‘Binpairs’ strategies with sequence alignment based binning methods

The effectiveness of considering pairing-information while binning with sequence alignment-based approaches is evident from Fig. 2, for both the short clones and long clones dataset. The correct and specific assignments by MEGAN-paired approach [3], are observed to be significantly better as compared to the conventional MEGAN approach [2]. The results obtained using ‘Binpairs’ strategies I, II and III (SI, SII and SIII) in combination with (conventional) MEGAN also provides similar number of correct and specific assignments as that by the MEGAN-paired approach. However, the number of wrong assignments by these three ‘Binpairs’ strategies is seen to be significantly lower as compared to that by MEGAN-paired approach. ‘Binpairs’ Strategy IV (SIV) fails to provide any improvement in the binning accuracy and a majority of the reads remain unassigned. It may be noted that this strategy was designed to be a conservative approach (see Methods), which records the individual taxon assignments for both reads constituting a pair, and subsequently (re)assigns both to the higher level (non-specific) taxon. The poor performance of SIV signifies that in most cases for these datasets, one of the reads constituting a mate-pair was originally binned to a higher (non-specific) taxonomic level, or remained unassigned.

The total number of correct assignments using ‘Binpairs’ strategies SI, SII and SIII in combination with the other two sequence alignment-based binning methods, namely, DiScRIBinATE and SOrt-ITEMS, is observed to be almost 1.5–2 times higher. However, in both these cases, SI strategy is seen to assigns highest number of reads at the specific levels of ‘genus’ or below. SII and SIII strategies, being slightly more conservative than SI (see Methods), is observed to assign more reads at the intermediate taxonomic levels of ‘family’ and ‘order’, but at the same time seen to have relatively lower number of incorrect assignments as compared to that by SI.

Overall, the results indicate that combining the ‘Binpairs’ strategies for utilizing pairing information (SI, SII and SIII) along with any of the alignment-based binning algorithms, provides significant improvements in the number of correct assignments. This gain in number of assignments is not at the expense of accuracy, as testified by the moderate proportions of ‘wrong assignments’ in the binning results utilizing SI, SII and SIII strategies. Out of all the combinations of ‘Binpairs’ strategies and alignment-based binning algorithms, SI when used in conjunction with DiScRIBinATE provides the highest number of correct assignments at specific levels.

### Validation of ‘Binpairs’ strategies with composition-based and hybrid binning methods

The ‘Binpairs’ strategies SI, SII and SIII, when used along with the composition-based method INDUS, and the hybrid binning methods, SPHINX and TWARIT, provide expected improvements in binning accuracy. Maximum number of assignments at specific taxonomic levels is obtained when SI strategy is adopted. The number of specific assignments after using strategies SII and SIII are also observed to be significantly better than the original accuracies of the SI and SII strategies, in combination with either of SPHINX or TWARIT or INDUS (Fig. 2), are at least at par or better than original binning results obtained with the (time binning outcome. Considering the number of incorrect assignments, it appears that SII and SIII exhibit an acceptable trade-off between the specific and wrong assignments, when used in conjunction with composition-based and hybrid binning methodologies. However, compared to the composition-based and hybrid approaches, the results obtained with alignment-based methods are seen to be relatively better. It may be noted here that in spite of exhibiting relatively better accuracy, the sequence alignment-based binning approaches are significantly more time consuming than the composition based and hybrid methods. Therefore, in cases where very high sequencing throughput is obtained (as expected with Illumina sequencers), the later methods can be deemed to be more appropriate for practical use. The time taken by different evaluated methods, for binning the simulated ‘Roche’ and ‘Illumina short-clones’ datasets are provided in 2. The hybrid and composition-based methods (viz., SPHINX, TWARIT and INDUS) are observed to be more than 25 times faster than the sequence alignment based approaches. Given that the binning accuracies of the SI and SII strategies, in combination with either of SPHINX or TWARIT or INDUS (Fig. 2), are at least at par or better than original binning results obtained with the (time consuming) alignment-based algorithms, these strategies can be appropriately adopted for binning large Illumina (paired-end) data-sets.

### Comparison with binning results of ‘Roche’ reads

Table 1 provides a comparison between the results obtained with simulated ‘Illumina’ (paired-end) reads (2×150 bp), using the ‘Binpairs’ strategies, and those obtained with a dataset consisting of relatively longer (∼400 bp) ‘Roche’ reads, drawn from the same simulated microbiome. The binning accuracy is expected to be better for longer ‘Roche’ reads as compared to shorter ‘Illumina’ reads. It is interesting to observe that by utilizing the pairing information, adoption of the ‘Binpairs’ strategies is observed to significantly improve binning accuracy, thereby making ‘Roche’ like binning results attainable even with smaller read lengths (Table 1). In order to further evaluate how binning results obtained after incorporation of ‘Binpairs’ strategies compare with those of a real metagenomics dataset sequenced with the Roche platform, a validation study was performed with metagenomic dataset from the oral metagenome project. S1 Fig. shows the different bacterial groups (cumulated at the phylum level) identified, along with their respective proportions, when the ‘Roche’ reads of the oral metagenome dataset was subjected to binning with SPHINX. The figure further depict the results obtained by binning the simulated ‘Illumina’ (paired-end) reads with ‘Binpairs’ Strategy II (in conjunction with SPHINX) for an easy comparison. The results indicate similar taxonomic profiles detected by the two different methods, thereby attesting the suitability of the suggested ‘Binpairs’ strategies for analysis of real metagenomics datasets.

**Table 1:** Comparison of taxonomic binning efficiency obtained with ‘Roche’ reads and ‘Illumina’ paired-end reads.

Type of reads	Binning strategy	Taxonomic assignments (%)				
		Genus andbelow	Family andOrder	Class andPhylum	Wrongassignments	Unassignedreads
Roche reads	MEGAN	24.4	7.9	10.4	28.5	28.8
Illumina paired-end reads	MEGAN+SIII	17.8	6.2	6.0	10.5	59.5
Illumina paired-end reads	MEGAN (paired)	18.0	4.9	7.3	18.5	51.3
Roche reads	DiScRIBinATE	20.9	15.8	17.9	9.5	36.0
Illumina paired-end reads	DiScRIBinATE+SI	22.5	8.9	11.7	9.0	47.9
Roche reads	SOrt-ITEMS	21.6	15.1	15.4	25.2	22.8
Illumina paired-end reads	SOrt-ITEMS+SI	20.6	6.2	9.5	22.5	41.1
Roche reads	INDUS	16.4	2.3	14.3	13.0	54.1
Illumina paired-end reads	INDUS+SIII	17.1	3.8	3.0	4.9	71.3
Roche reads	SPHINX	18.4	4.5	13.8	16.7	46.6
Illumina paired-end reads	SPHINX+SIII	16.6	3.3	3.0	2.8	74.3
Roche reads	TWARIT	22.0	4.2	7.8	11.4	54.7
Illumina paired-end reads	TWARIT+SIII	15.6	3.6	2.9	7.3	70.6

Roche reads have been binned using different existing binning algorithms. Illumina paired-end reads have been binned using a combination of ‘Binpairs’ strategies and existing algorithms. Results of representative ‘Binpairs’ strategies, which provided the best results, are illustrated in this table.

It is important to note here that, for all the validation sets used in this study, the volume of simulated sequence data in the ‘Roche’ and ‘Illumina’ sets are equivalent (see Methods). In a realistic scenario, the sequencing throughput from a Illumina sequencer is around 5–10 folds higher. It is therefore expected that sequencing a metagenome on an Illumina platform (using a ‘paired-end’ protocol), and subsequently using the discussed ‘Binpairs’ strategies of utilizing the ‘pairing’ information, will result in obtaining a deeper coverage of the metagenome with sufficient resolution of the taxonomic profile.

## Conclusion

The results of the present study suggest that utilizing the mate-pairing information for Illumina short reads can lead to significant improvements in taxonomic binning as compared to when each reads are binned independently. The improvements can be expected to produce comparable results to those achievable with longer Roche reads. Considering the cheaper costs and higher throughput of the Illumina platforms (as compared to other NGS technologies producing longer reads), the adoption of Illumina paired-end sequencing for metagenomics studies seems to be more than a feasible option. The ‘Binpairs’ strategies described in this paper for utilizing the mate-paired sequencing information are simple but effective, and don’t necessitate any significant modifications to the existing metagenomics protocols (either at the sequencing or the analysis stage).

## Supporting Information

S1 Fig **Evaluation of the ‘Binpairs’ strategy for a real metagenomic dataset.**
(PDF)

S1 Table **List of the 20 organisms used for constructing simulated test data sets.** For each test organism, the phylogenetic similarity status with respect genomic database (comprising of 952 complete microbial species) are also presented.
(PDF)

S2 Table **Time taken by different binning methods for taxonomic assignment of reads constituting the simulated ‘Roche’ and ‘Illumina short-clones’ datasets.**
(PDF)
