# Supplementary material for: Binpairs: Utilization of Illumina Paired-End Information for Improving Efficiency of Taxonomic Binning of Metagenomic Sequences
Source: PLoS One. 2014 Dec 31;9(12):e114814. doi: 10.1371/journal.pone.0114814 (PMC4281075; doi:10.1371/journal.pone.0114814)
Supplement: S1 Fig — Evaluation of the ‘Binpairs’ strategy for a real metagenomic dataset. (PDF) [file pone.0114814.s001.pdf]

Figure S1: Evaluation of the ‘Binpairs’ strategy for a real metagenomic dataset

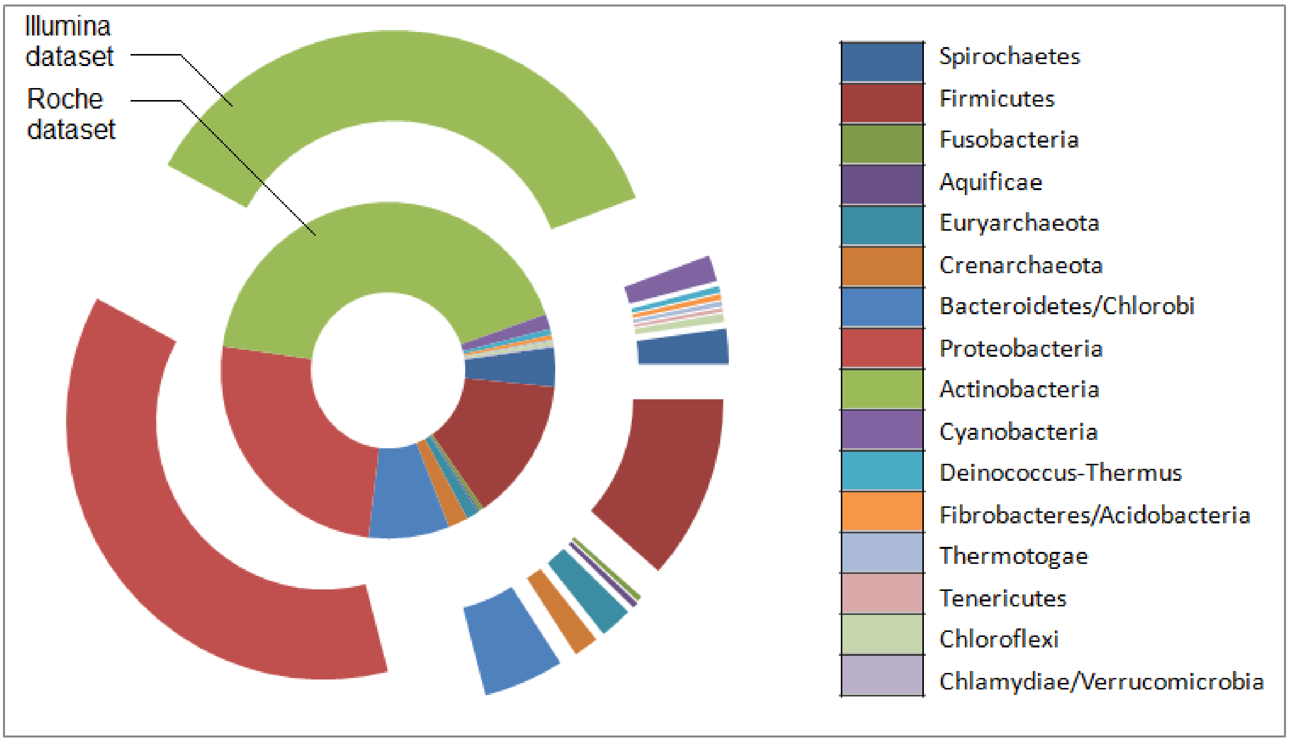

Comparison of binning results of the Oral metagenome dataset. The ‘Roche’ reads (~400 bp in length) constituting this metagenome were binned with SPHINX. Equivalent volume of simulated ‘Illumina’ mate-paired reads (see Methods) were binned using ‘Binpairs’ strategy SII in combination with SPHINX.
