# Supplementary material for: Binpairs: Utilization of Illumina Paired-End Information for Improving Efficiency of Taxonomic Binning of Metagenomic Sequences
Source: PLoS One. 2014 Dec 31;9(12):e114814. doi: 10.1371/journal.pone.0114814 (PMC4281075; doi:10.1371/journal.pone.0114814)
Supplement: S1 Table — List of the 20 organisms used for constructing simulated test data sets. For each test organism, the phylogenetic similarity status with respect genomic database (comprising of 952 complete microbial species) are also presented. (PDF) [file pone.0114814.s002.pdf]

Table S1: List of the 20 organisms used for constructing simulated test data sets. For each test organism, the phylogenetic similarity status with respect genomic database (comprising of 952 complete microbial species) are also presented.

Organism name	Nearest taxonomic relative in database
Caldisericum exile AZM16c01	at Phylum level
Coriobacterium glomerans PW2	at Order level
Marivirga tractuosa DSM 4126	at Family level
Oscillibacter valericigenes Sjm18-20	at Family level
Fluviicola taffensis DSM 16823	at Family level
Gardnerella vaginalis HMP9231	at Genus level
Halomonas elonga DSM 2581	at Genus level
Sulfuricurvum kujiense DSM 16994	at Genus level
Dickeya dadantii 3937	at Genus level
Helicobacter mustelae 12198	at Species level
Slackia heliotrinireducens DSM 20476	at Species level
Sulfurimonas autotrophica DSM 16294	at Species level
Thermus oshimai JL-2	at Species level
Arthrobacter arilaitensis Re117	at Species level
Pseudomonas aeruginosa NCGM2.S1	at Strain level
Akkermansia muciniphila ATCC BAA-835	included in database
Candidus Sulcia muelleri SMDSEM	included in database
Desulfobacca acetoxidans DSM 11109	included in database
Serratia proteamaculans 568	included in database
Xylella fastidiosa M12	included in database
