# Supplementary material for: Binpairs: Utilization of Illumina Paired-End Information for Improving Efficiency of Taxonomic Binning of Metagenomic Sequences
Source: PLoS One. 2014 Dec 31;9(12):e114814. doi: 10.1371/journal.pone.0114814 (PMC4281075; doi:10.1371/journal.pone.0114814)
Supplement: S2 Table — Time taken by different binning methods for taxonomic assignment of reads constituting the simulated ‘Roche’ and ‘Illumina short-clones’ datasets. (PDF) [file pone.0114814.s003.pdf]

Table S2:Time taken by different binning methods for taxonomic assignment of reads constituting the simulated 'Roche' and 'Illumina short-clones' datasets.

**Method**	**Total time taken for taxonomic assignments (in mins) ***	
	**Roche Dataset**	**Illumina 'short-clones' Dataset**
Megan	1292	1060
Megan-Paired	-	1145
Megan + SI	-	1063
Discribinate	1296	1061
Discribinate + SI	-	1064
Sort-items	1352	1080
Sort-items + SI	-	1083
Sphinx	68	40
Sphinx + SI	-	42
Twarit	16	12
Twarit + SI	-	15
Indus	64	36
Indus + SI	-	39

* Processing times of strategies SI, SII, SIII and SIV are equivalent. On a similar note, the processing time required for the 'short clones' and the 'long clones' dataset are also equivalent.
